# Respiratory adaptation to climate in modern humans and Upper Palaeolithic individuals from Sungir and Mladeč

**DOI:** 10.1038/s41598-021-86830-x

**Published:** 2021-04-12

**Authors:** Ekaterina Stansfield, Philipp Mitteroecker, Sergey Y. Vasilyev, Sergey Vasilyev, Lauren N. Butaric

**Affiliations:** 1 Unit of Theoretical Biology, Department of Evolutionary Biology, University of Vienna, Althanstrasse 14, 1090 Vienna, Austria; 2 Moscow State University of Medicine and Dentistry, Moscow, Russian Federation; 3 Institute of Anthropology and Ethnography, Moscow, Russian Federation; 4 Department of Anatomy, College of Osteopathic Medicine, Des Moines University, Des Moines, USA

**Keywords:** Ecology, Evolution

## Abstract

As our human ancestors migrated into Eurasia, they faced a considerably harsher climate, but the extent to which human cranial morphology has adapted to this climate is still debated. In particular, it remains unclear when such facial adaptations arose in human populations. Here, we explore climate-associated features of face shape in a worldwide modern human sample using 3D geometric morphometrics and a novel application of reduced rank regression. Based on these data, we assess climate adaptations in two crucial Upper Palaeolithic human fossils, Sungir and Mladeč, associated with a boreal-to-temperate climate. We found several aspects of facial shape, especially the relative dimensions of the external nose, internal nose and maxillary sinuses, that are strongly associated with temperature and humidity, even after accounting for autocorrelation due to geographical proximity of populations. For these features, both fossils revealed adaptations to a dry environment, with Sungir being strongly associated with cold temperatures and Mladeč with warm-to-hot temperatures. These results suggest relatively quick adaptative rates of facial morphology in Upper Palaeolithic Europe.

## Introduction

The presence and the nature of climate adaptation in modern humans is a highly debated question, and not much is known about the speed with which these adaptations emerge. Previous studies demonstrated that the facial morphology of recent modern human groups has likely been influenced by adaptation to cold and dry climates^1–9^. Although the age and rate of such adaptations have not been assessed, several lines of evidence indicate that early modern humans faced variable and sometimes harsh environments of the Marine Isotope Stage 3 (MIS3) as they settled in Europe 40,000 years BC^10^. In the present study, we explore whether the facial morphology of two of the earliest Upper Palaeolithic humans from Europe, Mladeč-1 (Czech Republic) and Sungir-1 (Vladimir, Russia), demonstrate signatures of adaptation similar to that of boreal-to-temperate-adapted modern human groups, as suggested by their paleo-climatological context.

###  Evolution of craniofacial diversity

Current patterns of modern human craniofacial diversity evolved by a combination of both neutral and selective processes. Connections between craniofacial morphology, climate, as well as genetic and geographical similarities have long been investigated by various multivariate statistical approaches^1–8,11–13^. Several of these studies indicated that the neurocranium and upper face partly track neutral genetic and molecular distances, while midfacial shape also reflects climate^3,11^. However, Betti et al.^5^ claimed that neutral processes were more important than climate in shaping the overall human cranium. In their point of view, a large proportion of the signal for climate-related natural selection is due to the inclusion of populations from extremely cold regions (i.e., Arctic samples). Along these lines, von Cramon-Taubadell^14^ reported that functional modules in the cranium are highly correlated with genetic distances between populations. Finally, and contrary to Harvati and Weaver^3^ and Smith^11^, Reyes-Centeno et al.^12^ suggested that the temporal bone and the face exhibited stronger associations with genetic distances than the neurocranium.

Other studies pointed to strong climatic influences on midfacial structure, particularly those of the internal nasal region. Evteev et al.^15^ reported a strong association of mid-facial morphology in Asian groups with climatic variables that contrast the temperate climate of East Asians and the very cold-dry climate of North Asians. However, when genetic distances between groups were accounted for, these correlations reduced. In line with Evteev et al.^15^, Maddux et al.^8^ demonstrated that the internal nasal cavity exhibited an ecogeographic distribution consistent with climatic adaptation, with crania from colder or drier environments displaying internal nasal cavities that are longer, taller, and narrower (especially superiorly) compared to those from hotter and more humid environments. Clinical and experimental studies suggest that tall, narrow noses assist in thermoregulatory processes to warm and humidify the inspired air to protect lung tissues from desiccation^8^. Furthermore, cold-dry adapted crania tend to display tall zygomatic-maxillary interfaces, with taller and wider maxillary sinuses^16,17^. No strong correlations between maxillary sinus size and climatic conditions have been shown, but current literature suggests that sinuses act as zones of accommodation for ontogenetic and phylogenetic changes of surrounding craniofacial structures, including the nasal cavity^17–20^.

Overall, the discussion about the significance of climate for modern human variation and adaptation is still unresolved, partly owing to methodological challenges and misconceptions. For instance, correlations between neutral genetic distances and measures of morphological dissimilarity (morphometric distances) do not necessarily indicate a neutral mode of evolution, as is often assumed in the literature reviewed above. Both neutral genetic markers as well as climate are more similar in geographically adjacent populations than in more distant populations; climate adaptation would thus also lead to a correlation between neutral genetic and morphometric distances^21^. Moreover, multivariate morphometric distances pool all measured traits, which may have very different functional roles and evolutionary dynamics. Correlations with such multivariate distances may thus lack the statistical power necessary for identifying signatures of adaptation. Nevertheless, it is becoming clear that the mid-face (particularly nasal morphology) is more strongly associated with climatic variables than the neurocranium.

It can be expected that changes in nasal morphology were particularly important in helping our African ancestors to settle in the colder and drier environments of Europe during the Late Pleistocene. Indeed, several recent empirical studies suggest that Neanderthal nasal morphology was compatible with the need for warming and humidifying inhaled air^22,23^. Evteev et al.^7^ also showed that several Upper Palaeolithic Europeans were similar to modern North-East European groups in terms of mid-facial morphology. However, these researchers did not extend their study to directly compare the Upper Palaeolithic European samples to modern North Asian samples, which may be more reflective of cold-adapted morphologies^6^. Furthermore, it is unclear as to when these nasal adaptations developed in the modern human lineage. The Mladeč-1 and Sungir-1 specimens represent two well-preserved crania of early Upper Paleolithic individuals, who lived in relatively harsh environments and may thus shed light on the question of climate adaptation in early Europeans.

### Mladeč and Sungir: geographical, temporal, and climatic context

The Mladeč and Sungir sites are well known owing to the numerous individuals whose remains were buried or otherwise deposited at these sites. First excavated in 1955, the Sungir site (56° 10′ 30″ N, 40° 30′ 30″ E) is located near the city of Vladimir, about 192 km northeast of Moscow, Russia^24^; excavations at this site have unveiled several graves and at least nine individuals^25,26^. The Sungir remains have been recently dated to around 35,000 ^14^C years BP^25^. Sungir-1, an almost complete skeleton of an older male, was unearthed in 1964 from Grave 1 with numerous grave goods including ochre, ivory beads, and other types of body ornamentation^24,26^.

The Mladeč caves are located in central Bohemian massif, Olomouc Krai of the Czech Republic, and yielded remains of up to 10 individuals^27^. This site was largely excavated under the direction of Szombathy in the late 1880s; excavations continued under various researchers through the mid-1900s. The Mladeč-1 cranium was unearthed from the main cave in 1881^28^. Note that earlier reports assigned the Mladeč-1 cranium as male, but it could also belong to a younger female^29^. Direct radiocarbon dates for Mladeč human material cluster around 31,000 ^14^C uncalibrated years before present^27^.

Genetic studies of the Sungir remains have clearly shown their close relationship with modern Europeans to the exclusion of all East Asian groups^30^. Although there is no direct evidence of a close genetic kinship between Sungir and Mladeč, Sungir belongs to the same genetic pool as Dolní Věstonice, Gravettian people of Moravia^30^. Mladeč comes from a neighbouring Moravia geographical region and immediately predates Dolní Věstonice people. Given that previous archaeological and palaeo-biological accounts suggest population continuity between the late Aurignacian people of Mladeč and Gravettian people of Dolní Věstonice^31^, we assume that Mladeč and Sungir took ancestry in the same wave of migrations out of Africa.

Roughly contemporaneous, several lines of evidence suggest that the Mladeč and Sungir inhabitants also lived in broadly similar climatic conditions. During MIS 3, the neighbouring Mladeč Moravian area was partly covered by woodland dominated by conifers with the accompaniment of some deciduous trees, including oak, beech, and yew^32,33^. At the same time, geological studies of the frost features and molluscs biodiversity suggests a cold subarctic tundra^34^, thus pointing to a variable and changing environment that ranged from the steppe, shrub and steppe, and partially forested landscape^33^. This picture is repeated at the Sungir site, where soil and pollen analyses point to the Bryansk interstadial (32–24 kyBP), a slightly warmer period in the MIS 3, where boreal vegetation was dominated by spruce and pine with the admixture of birch^35–37^. Thus, both individuals likely lived in environmental conditions that could be similar to, but more continental than at present, with sharper changes between warm and cold periods as in modern South Eastern Siberian regions and Mongolia^38–40^.

As such, several studies have assessed how the morphology of the Sungir specimens compares to other peri-glacial hominins, and whether morphological changes indicative of climatic adaptation are evident^7,26,41–43^. Bunak^41^ found that Sungir-1 is morphologically most similar to early central Europeans, including Predmostí 3 and other Cro-Magnon-like specimens. In a more recent study, Evteev et al.^7^ also found close similarities between Sungir-1 and Late Holocene modern Europeans. While informative, these studies may be considered limited in that they focus on external cranial features, without including internal features of the nasal cavity (such as internal nasal height and breadth) that are more functionally related to climate^8^.

### Aim of the study

The purpose of this study is to first investigate climate-related variation in upper respiratory organs (i.e., the nose and maxillary sinuses) within a world-wide sample of modern human groups. We then assess whether two Upper Palaeolithic individuals, Mladeč-1 and Sungir-1 (hereby referred to simply as Mladeč and Sungir), display mid-facial and respiratory features characteristic of modern groups adapted to the boreal-to-temperate climate, as suggested by the paleo-climatological association of the fossils. Given the close geographical and temporal proximity as well as possible common genetic ancestry, we expect the two fossils to display similar climatic adaptations.

We placed 61 three-dimensional landmarks on the external and internal mid-face of the two fossils and of 233 Holocene human crania from diverse ecogeographic regions, and analysed the data using geometric morphometrics. Most previous studies pooled different cranial features and did not take into account autocorrelations of morphological and climate variables resulting from the geographical proximity of human groups. Here, we rectify this problem by introducing a weighted reduced rank regression approach (see Methods).

## Results

### Reduced rank regression of mid-facial shape

We analysed the shape and size of the mid-face separately. The association between the average face shapes of the groups and the four climate parameters was explored by reduced rank regression (RRR). Similar to the more familiar two-block partial least squares analysis (PLS), RRR represents the multivariate association between two blocks of variables (here, shape and climate) by several pairs of linear combinations, or latent variables, of the measured variables. Unlike PLS, RRR distinguishes between a dependent and an independent block and maximizes the regression slope of one linear combination on the other, i.e., the effect of one unit change of the independent variables on the dependent variables, regardless of the occurring variance. Hence, RRR also captures effects of climatic properties on facial shape even if they vary little in the sample. In our case, the regression slopes were also corrected for the autocorrelation due to geographic proximity (see Methods for more details).

**Figure 1:**
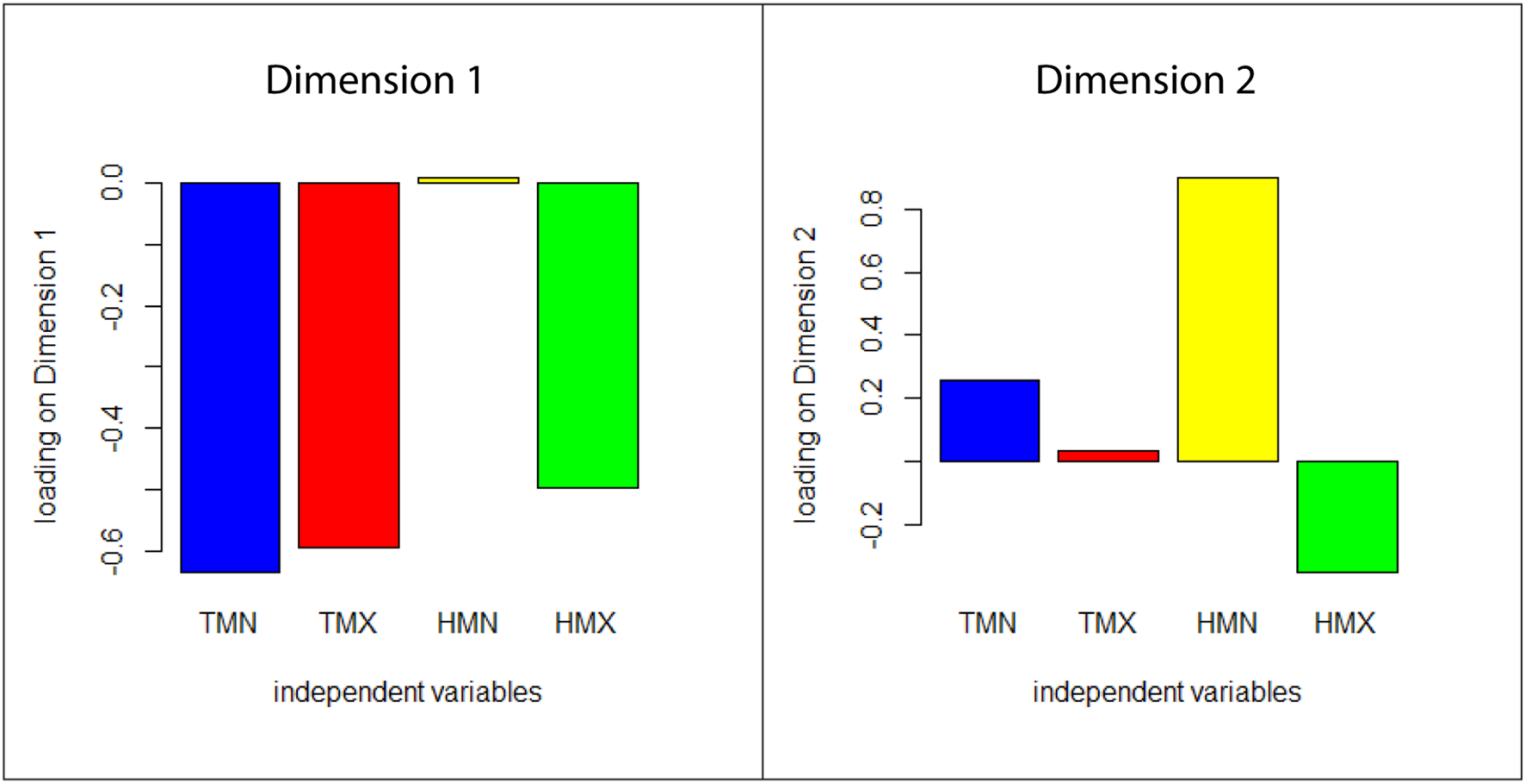
Climate loadings for the first two dimensions of reduced rank regression (RRR). The first RRR dimension accounts for 73% of the total association between the four climate variables and facial shape (i.e., 73% of the summed squared partial regression coefficients), and the second dimension accounts for 21%. TMN—average minimum temperature of the coldest month; TMX—average maximum temperature of the hottest month; HMN—average minimum humidity (vapour pressure) of the least humid month; HMX—average maximum humidity (vapour pressure) of the most humid month. All climate variables were transformed to *z*-scores.

The climate variables explained 54.5% of the variance in facial shape among recent modern human groups (corrected for geographic distances; see Methods). Of this accounted variance, over 94% was summarized by the first two dimensions of the RRR (Supplementary Table S1). The first dimension had a strong negative loading on both temperature variables and on maximum humidity. The second dimension mainly loaded on minimum humidity. In other words, the first dimension described the association of facial shape with cold and dry climate while the second dimension described the influence of humidity during the driest months (Fig. 1).

**Figure 2:**
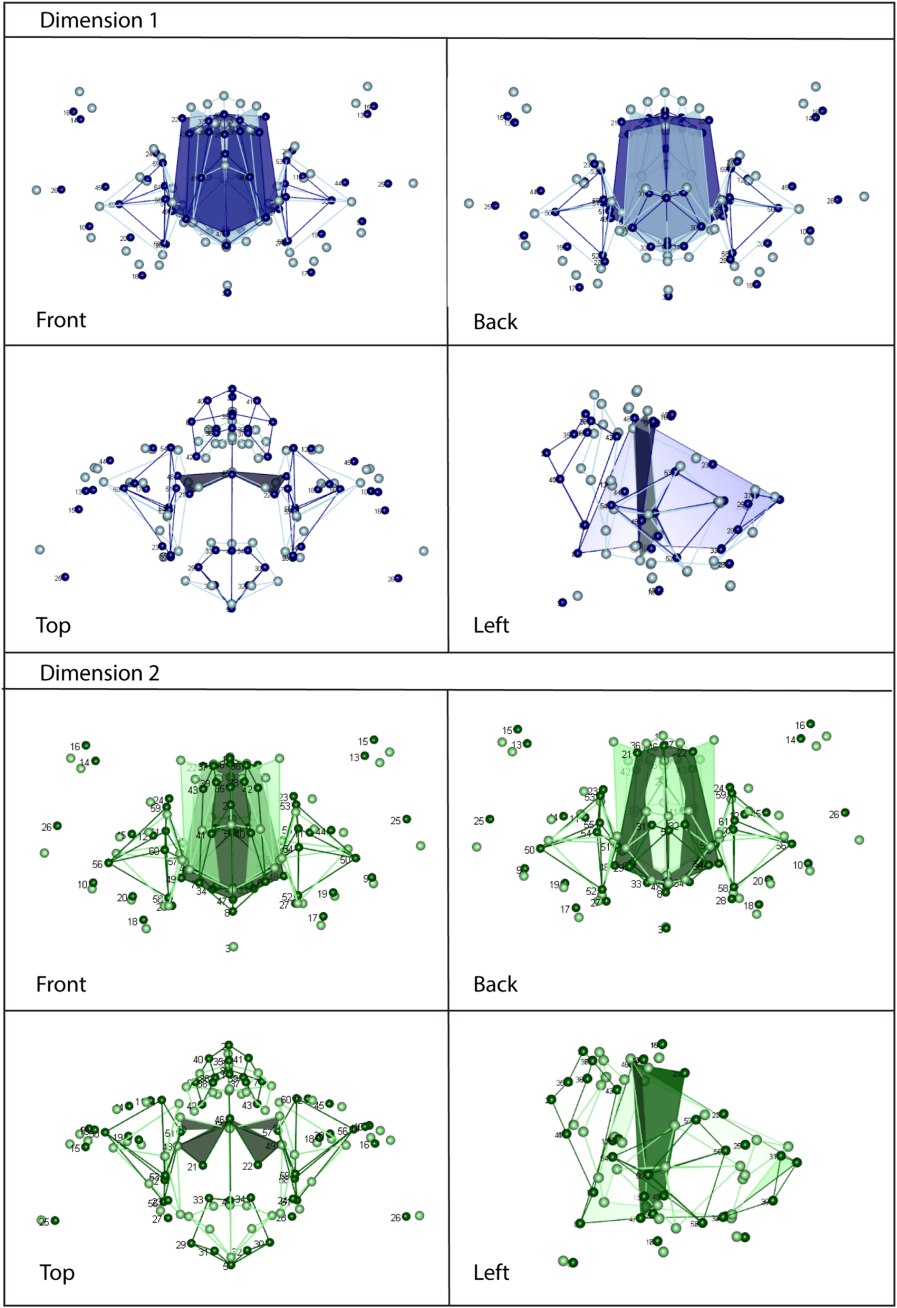
Face shape described by the first dimensions of the reduced rank regression. Dimension 1: Dark blue wireframes correspond to warmer climates and light blue wireframes to colder climates. Dimension 2: Dark green wireframes correspond to drier climates and light green ones to more humid climates. In both cases, shaded surfaces represent the internal nose configuration.

As in other geometric morphometric contexts, the loadings of the shape vectors can be visualized as shape deformations (Fig. 2). The first dimension corresponded to the relative width of the external and internal nose and the relative size of the sinuses. In other words, populations living in warmer and more humid climates tend to have relatively wider and more prominent external noses, wider and lower internal nasal structures and overall smaller sinuses, all in combination with a slightly narrower and lower mid-face. A colder and drier climate, by contrast, is associated with an overall taller mid-face, narrower and flatter external nose, tall internal nose and large sinuses (Fig. 2). Along the second dimension, populations from drier climates, as reflected by minimum humidity, tend to have taller faces with “high cheek-bones”, a narrow but prominent external nose, and a narrow internal nose with slightly more posterior s*uperior ethmoidale* points. In dry climates, the maxillary sinuses are shifted anteriorly compared with their position in more humid climates.

**Figure 3:**
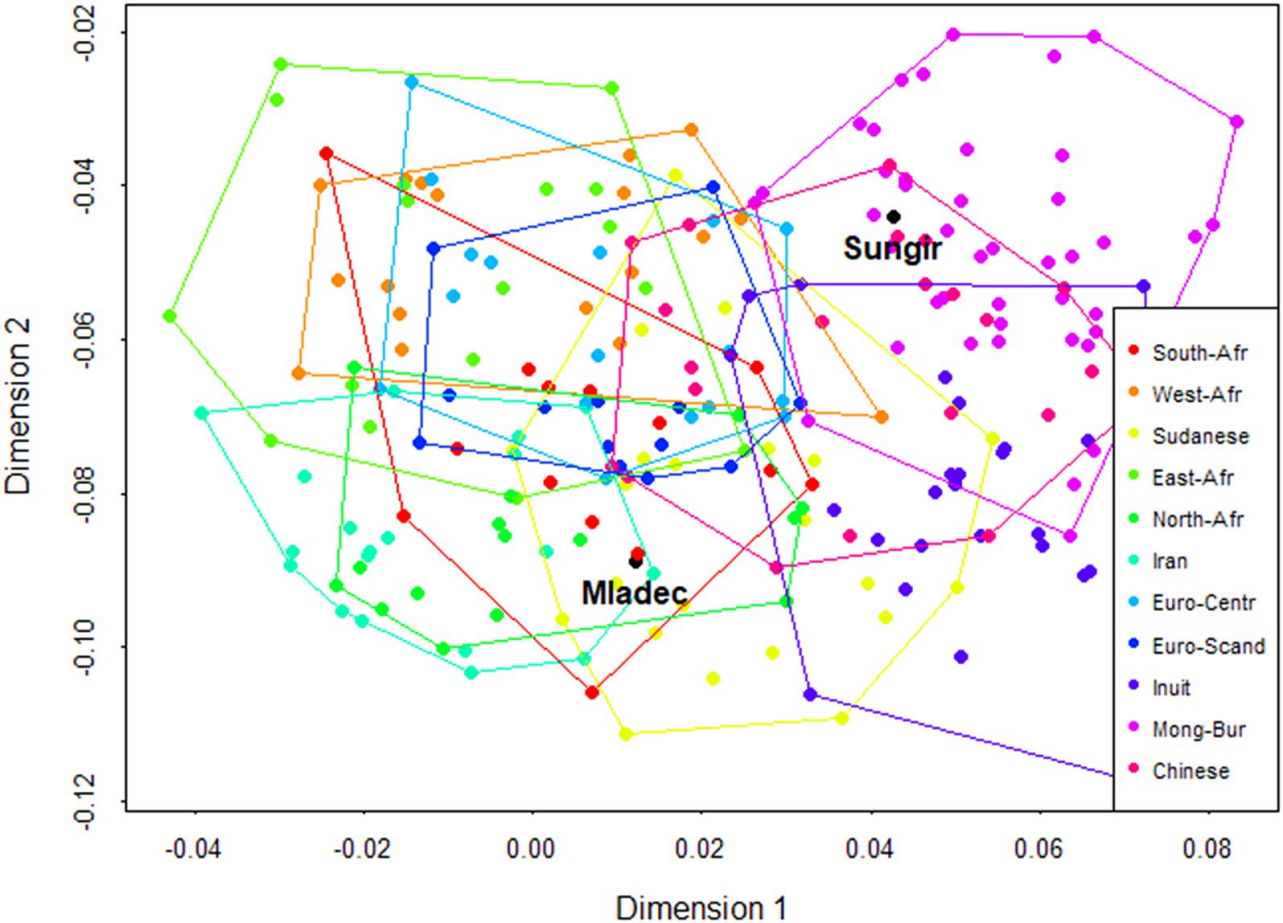
Individual shape configurations projected into the space of the first two shape dimensions of the RRR. Dimension 1  accounts for 73% and Dimension 2 for 21% of the shape variation related to climate.

These two RRR dimensions accounted for about 94% of the shape variation that relates (linearly) to variation in climate. The two corresponding dimensions for the shape coordinates thus represent the shape features that are most affected by climate. When projecting the individual shape configurations onto these two vectors, they span an “adaptive shape space,” in which we ordinate the individual shape variation that is maximally influenced by climate (rather than by other environmental factors or population history). In this space, Mongolian-Buryats and Inuits scored highest along the first loading vector, in agreement with their origin from cold and arctic climates. Africans and Europeans clustered in the warm-to-temperate end of the first vector but were subdivided along the second vector into groups from climates with lower and higher humidity of the driest month, respectively. Here, Sudanese and Iranians were associated with dry climates, as were North-Africans. In agreement with their very dry climate, Inuits scored among the dry-adapted groups along both dimensions (Fig. 3).

This ordination differs considerably from that achieved by principal component analysis (PCA) or canonical variate analysis (CVA), which were dominated by population history; these analyses grouped Alaskan Inuits together with other East Asian groups and placed both Sungir and Mladeč in proximity to European samples (see Supplementary Fig. S1). In the adaptive shape space, by contrast, the fossil specimens were surprisingly different from each other (Fig. 3). Sungir fell into the distribution of Mongolian-Buryats, suggesting an adaptation to the colder climate with smaller *maximum* humidity. Mladeč plotted with Iranians and African groups from warmer climates with low *minimum* humidity.

### Centroid size

**Figure 4:**
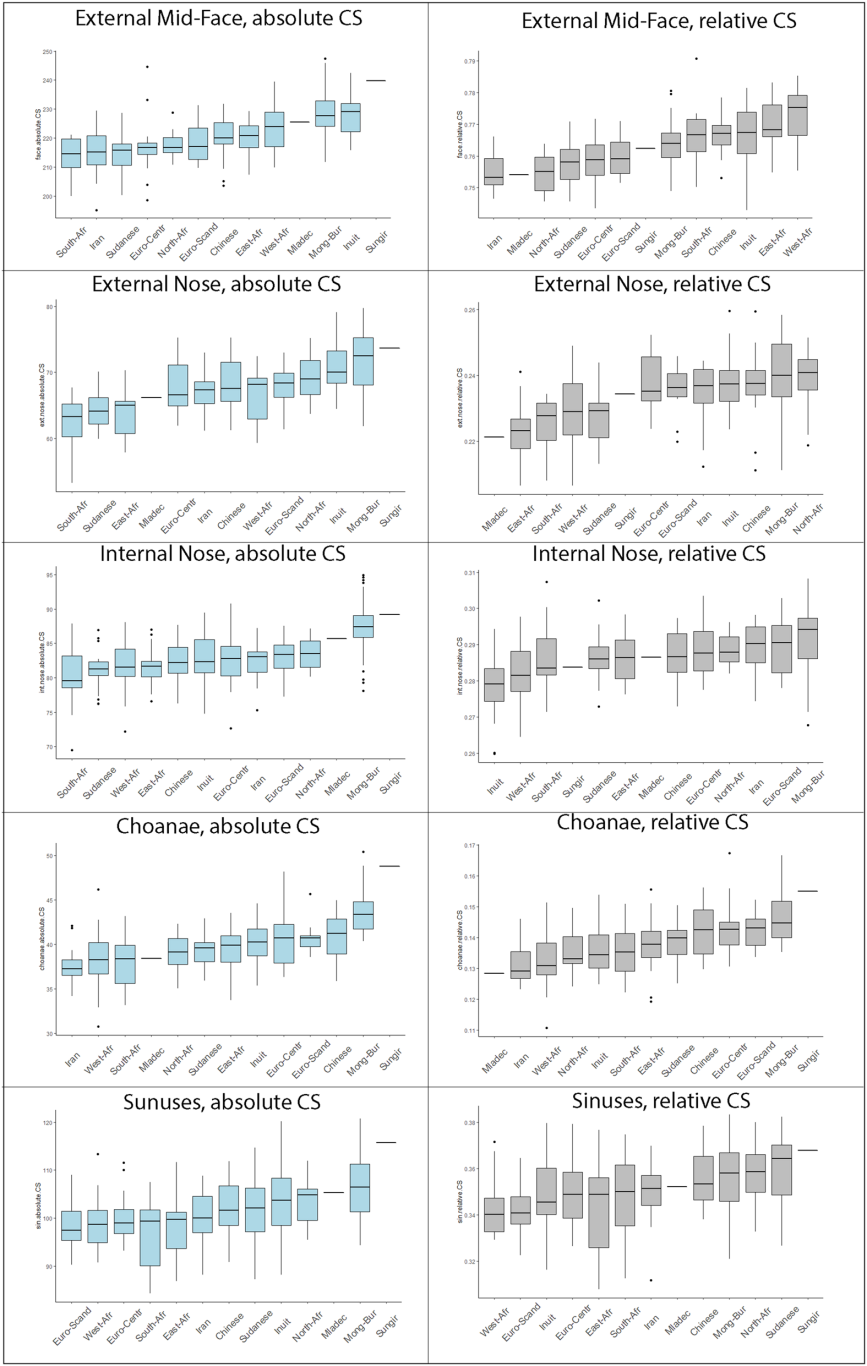
Absolute and relative centroid size for different anatomical regions. The groups in the boxplots are sorted by the median centroid size in ascending order.

Figure 4 compares the absolute sizes (as measured by centroid size, CS) and the relative sizes of the mid-face and of the upper respiratory regions among the sampled groups (relative sizes were computed by dividing the CS of the regions by the CS of all 61 landmarks). The largest faces were found among the East Asian groups, especially in Mongolian-Buryats and Inuits. The external nose was particularly large in groups from colder climates, such as Mongolian-Buryats, Inuits and Scandinavians. The internal nose provided a mixed picture, with a larger size found mostly in groups from dry climates (Mongolian-Buryats, Scandinavians, Iranians). The choanal region provided only a weak association with climate. The largest choanae were found among the cold-adapted Mongolian-Buryats, while small choanae were mostly found in groups from warmer areas (as in Iran, West and South Africans). Sinuses were largest in Mongol-Buryats and Inuits, who come from dry and cold climates. North Africans were more similar to groups from dry climates despite relatively high values of minimum and maximum humidity in Egypt throughout the year (Table 1). Linear regressions of CS on the climate variables showed that minimum temperature and, to a lesser extent, maximum humidity are the two main factors that explain the trend in the absolute CS (Supplementary Table S2). At the same time, the relative size of the upper respiratory structures did not show clear climate-related trends (Supplementary Table S2).

**Table 1:** Groups and individuals in the study.

Individuals		n	Geographic location*			Temperature (°C)		Water vapor pressure (HPa)	
			Place	LAT	LON	TMN	TMX	HMN	HMX
Sungir	Sungir1	1	Vladimir, Russia	56.2	40.3	− 13.53	24.04	2.64	15.29
Mladeč	Mladeč	1	Dĕtcovice, Czech Republic	49.42	17.1	− 4.05	24.46	4.49	14.69

Group	Subgroups	n	Place	LAT	LON	TMN	TMX	HMN	HMX
Chinese	Chinese^a^	22	Guangzhou, Guangdong Province	23.13	113.25	9.63	32.48	11.36	31.56
Inuits	Barrow Quad^a^	9	Point Barrow, Alaska	71.40	− 156.48	− 30.37	8.28	0.44	7.85
	Tigara Village^b^	20	Point Hope, Alaska	68.35	− 166.80	− 25.35	13.69	0.51	8.83
Mong-Bur	Buryats^a^	14	Nr. Lake Baikal	52.28	104.32	− 24.32	24.87	1.20	15.14
	Mongolians^a^	32	Mongolian Urga	47.92	106.91	− 28.87	23.02	0.63	11.17
Iran	Tepe Hissar^c^	19	Damghan, Iran	36.16	54.39	− 4.99	32.73	2.94	10.01
Euro-Centr	Former Yugoslavia^b^	8	Dubrovnik, Croatia	42.64	18.11	− 0.92	27.38	5.47	14.99
	Germany^c,d^	7	Frankfurt	50.11	8.68	− 2.88	24.04	5.79	14.40
	Netherlands^a,d^	2	Amsterdam	52.38	4.90	0.41	21.28	6.74	15.41
Euro-Scand	Scandinavia^c,d^	10	Nr. Falun, Sweden	61.33	15.27	− 12.39	20.04	3.24	12.11
North-Afr	Egyptians^a^	15	Cairo	30.06	31.24	18	35	10	22
Sudanese	Nubians^e^	22	Northern Dongola	18.28	30.38	10	43.5	6.5	14.5
East-Afr	Kenyans^a^	18	Nairobi	− 1.30	37.5	11.95	28.32	13.59	17.34
West-Afr	Côte d’Ivoire^a^	9	Côte d’Ivoire	6.50	− 4.7	20.95	34.12	24.59	28.54
	Gabon^a^	9	Gabon	− 0.72	8.78	20.98	30.92	24.52	30.27
South-Afr	Khoisan^b^	14	Kareeberg, Northern Cape	− 30.56	22.94	− 0.2	31	5	12

*LAT* Latitude, *LON* Longitude, *TMN* Average minimum annual temperature of the coldest month, *TMX* Average maximum annual temperature of the hottest month, *HMN* Average minimum annual water pressure, *HMX* Average maximum annual water pressure.

*Location coordinates are transferred into a decimal system.

^a^Smithsonian Institute, Washington DC.

^b^American Museum of Natural History, New York City, NY.

^c^University of Pennsylvania Museum of Archeology and Anthropology, Philadelphia through the Open Research Scan Archive.

^d^University of Leipzig Anatomical Collection, through NESPOS.

^e^Kulubnarti Collection, University of Colorado Boulder, CO.

In comparison with modern humans, Sungir and Mladeč are large individuals, whose mid-face resembles that of Mongolian-Buryats and Inuits in absolute size. The absolute size of the external nose differs between the two fossils, placing Mladeč among populations with warm to hot climates and Sungir among groups from cold and dry environments. Both, Sungir and Mladeč, have an absolutely large internal nose, as in many dry-adapted groups, but their relative sizes are small to intermediate. The absolute and relative size of the choanae associate Sungir with Mongolian-Buryats at the higher end, while Mladeč aligns with groups displaying smaller choanae (e.g., Iranians, North Africans). Both fossils have large maxillary sinuses, but sinuses in Sungir are also large relative to the size of its face. Overall, the absolute size of the respiratory organs place Sungir among groups from cold and mostly dry climates and Mladeč among groups from warmer climates.

## Discussion

Our results demonstrate that multiple aspects of internal and external mid-facial shape are associated with climate, even when accounting for spatial autocorrelation among groups. Temperature and minimum humidity were the main factors in driving climate-related variation in the shape of the upper respiratory tract. Groups from colder and drier climates tended to have an overall taller mid-face, a narrower and flatter external nose, a tall internal nose and large sinuses. They also tended to have a larger absolute size of the face and the upper respiratory tract. Groups from warmer regions, irrespective of their geographical origin, had a relatively wider and more prominent external nose, wider and lower internal nasal structures and overall smaller sinuses, all in combination with a slightly narrower and lower mid-face. The association of the mid-facial and respiratory morphology with minimum humidity was not as strong as that with temperature and maximum humidity.

Our results correspond with and develop on the previous accounts of climate-related trends. The morphology of the internal nose has been previously shown to correlate with humidity and to reflect environmental adaptation better than other parts of the respiratory system^8,44^, in agreement with the clinical and experimental literature^45,46^. Our study confirmed that populations in colder climates tend to have a relatively taller and narrower internal nose (Fig. 2). The dryness of the climate, irrespective of temperature, was reflected in a narrower superior part of the internal nasal cavity with a posterior positioning of the left and right *superior ethmoidale* points (also see^47^). Furthermore, the absolute, but not relative, size of the internal nose separated groups from drier and more humid climates, as also reported by Maddux et al.^8^.

In agreement with the observations by Buck^48^ and Butaric^49^, the absolute size of the maxillary sinuses did not clearly relate to climate (Fig. 4). However, the *relative* sinus dimensions as represented by the RRR shape score were larger in groups from cold climates (Fig. 2: Dimension 1). Maddux and Butaric^17^ suggested that the form of the internal nasal cavity and the sinuses are largely determined by the shape and size of the maxillary and zygomatic bones. Hence, future studies on maxillary sinus morphology should consider this structure in conjunction with the surrounding internal and external anatomy.

Among the four climate variables, minimum temperature had the strongest effect on the absolute size of the mid-face and the respiratory organs (Fig. 4, Supplementary Table S2). In contrast to Noback et al.^6^, we found that—unlike for overall face shape—the relative sizes of the separate respiratory organs did not consistently relate to climate. This inconsistency between absolute and relative sizes may be due to differential constraints imposed by heat retention versus those by the demands for warming and humidification of the inhaled air^45,46,50^.

### Climate-associated features in Sungir and Mladeč midfacial anatomy

Based on their midfacial morphology, several previous studies aligned Mladeč and Sungir with European groups^7,41,47^. Our results, however, show that the two fossils differ in climate-related features. This discrepancy is due likely to the different statistical and morphometric methods. Morphological similarities among populations are shaped by many developmental and evolutionary factors, including evolutionary drift and population history, which often dominate morphometric distances and ordinations (such as the CVA in Fig. S1). To circumvent this problem, we first identified the features of face shape that are most affected by climate in the worldwide sample, and then calculated individual scores for these “adaptive” features (Fig. 3). In other words, we quantified morphological similarities of Mladeč and Sungir only for the supposedly climate-adapted features, not overall shape. At the same time, these multivariate scores are expected to trace adaptive signatures more reliably than univariate traits because climate adaptation is likely to have only small effects on each single trait; when summed over multiple traits, however, the adaptive signature should stand out^51^. Additionally, spatial autocorrelation can induce spurious associations between morphology and climate, but, on the other hand, climate *is* geographically patterned. As we used a weighted least squares approach to correct for spatial autocorrelation, the presented results are very unlikely to reflect geographical patterns or population history only, but they could underestimate the actual adaptive signal.

Based on this approach, Sungir showed adaptation to cold climate due to a taller mid-face, a narrower and relatively flatter external nose, a tall internal nose and relatively large maxillary sinuses and choanal regions (Dimension 1, Figs. 2, 3). The absolute size of the mid-face and respiratory features aligned Sungir with Mongolian-Buryats, a group from a colder and drier climate, which is in agreement with studies describing this fossil’s morphology as ‘currently found only between Siberian mongoloids, Inuits and some American Indians’^52, p. 148]^.

However, it is important to note that thermoregulatory processes are not the sole function of the nose. Nasal size—particularly in terms of internal height dimensions and the choanal region—is largely driven by oxygen demand^8,53–55^. As metabolic rates increase in colder climates^23,56,57^, concomitant increases in oxygen demand may result in the relatively larger size of the internal nose and the choanae^23,54,56,57^.

Previous studies note that the Sungir individuals, particularly Sungir-1, were tall compared to later human groups^26,42^. While longer limb proportions for these fossils may point to an origin from hot climates see^58,59^, Sungir individuals also express traits of a prolonged growth period thanks to large marrow cavities in long bones and a capacious chest^42^ similar to Neanderthals and high-altitude populations^60^. The capacious posterior nasal choanae of Sungir found in the current study^26^, further support a scenario of nasal adaptation to a life of high-oxygen demand in cold-dry environments^54^.

Mladeč showed features of adaptation to a warmer environment along the first RRR dimension (relatively wide internal and external nose, relatively small maxillary sinuses; Figs. 2, 3). Additionally, Mladeč showed adaptation to a dry climate with “high cheek-bones,” a prominent external nose, and an internal nose with a slightly more posterior position of s*uperior ethmoidale* points, similar to African groups from dry climates as well as to Inuits (RRR dimension 2, Figs. 2, 3). The centroid size of the choanae placed Mladeč among groups from warmer climates. This contrasts with the absolute size of the mid-face, the internal nose and sinuses, which in Mladeč were still as large as in Mongolian-Buryats.

We set out with the expectation of similar climate adaptations in Sungir and Mladeč because the paleo-geographical and palynological evidence from their burial sites suggest a broad similarity of the climatic conditions experienced by these Upper Palaeolithic humans in about 30,000 y BP. The results of our analysis contradicted this expectation. An explanation of this discrepancy may come from the paleo-geographical evidence itself. During the MIS 3 interstadial, Moravia and the Mid-Russian European Plain experienced a range of conditions, with vegetation oscillating from tundra to forest, and with mammalian fauna spanning from open landscape species, such as woolly mammoths, to wolverines and ground squirrels, which prefer more wooded environments^32,61,62^. In addition, there is evidence of several sharp climate oscillations during the middle-interglacial that have been traced throughout Western and Eastern Europe^63,64^. In Dolní Vĕstonice, the climatic oscillations were evident from the palaeosoil stratigraphy and associated malacofauna^65^. In the Mid-Russian European Plain the evidence also comes from the palaeosoils and associated vegetation^62^. During these oscillations, colder and drier tundra conditions were followed by warmer periods with slightly more humid environments that encouraged the development of broad-leaf tree cover and associated malacofauna^66,67^. The warmer time periods may account for the adaptation to a warmer climate in Mladeč. However, it is also possible that Sungir’s ancestors had longer time in the periglacial environment and therefore more generations to adapt to the local climate than had the ancestors of Mladeč. This would imply a more complex pattern of human dispersal in the Upper Palaeolithic Eastern Europe, the evidence of which is still awaited. In either case, Sungir was already adapted to the cold and dry climate 35–31 ky BP.

The association of Sungir with modern groups from cold-dry environments and of Mladeč with the groups from a warmer and dry climate regarding their functional nose shape, irrespective of the overall morphological similarity between the two fossils (Supplementary Fig. 1), incites the question about the relatively high rate of facial and respiratory adaptation to the changing climate in the Upper Palaeolithic. To answer this question, further investigations with a wider sample of modern and fossil humans are required—particularly to sort out morphological differences among cold-dry versus hot-dry conditions and to analyze corollary effects, such as the relationship between metabolic rate, temperature, and upper respiratory morphology. Such studies will provide a better understanding of how our ancestors could have adapted to different environments, the timing in which these adaptations occurred, and how researchers can best infer this from the fossil record.

## Materials and methods

To assess both external and internal craniofacial morphology, we used computed tomographic (CT) scans of Sungir and Mladeč as well as several modern human (Late Holocene) samples from a wide range of climatic zones: Central Europeans (Germany, Bosnia, Croatia, Netherlands; n = 17), European Scandinavians (n = 10), Iranians (Tepe Hissar, Iran; n = 19), North Africans (Egyptians; n = 15), Sudanese (Nubians; n = 22), East Africans (Kenyans; n = 18), West Africans (Côte d’Ivoire, Gabon; n = 18), South Africans (Khoisan; n = 14), Central-North Asians (Mongolians and Buryats; n = 48), Southern East Asians (Southeast Chinese; n = 22), and Arctic Inuits (Barrow Quad and Point Hope, Alaska; n = 28). The geographic location, specific samples, climatic variables, and collection archives for these groups are presented in Table 1. Additional details regarding these samples can also be found in the previous works of author LNB^17,49,68^.

The Sungir CT scan was obtained via a Brilliance 64 (Philips, Netherlands) in Moscow, Russia, with voxel height and width of 0.51 mm × 0.51 mm, and a slice thickness of 0.3 mm. The Mladeč CT scan was obtained from the Digital @rchive of Fossil Hominoids (http://www.virtual-anthropology.com/3d-data/data-webshop/). This specimen was originally scanned in 1996 with a Phillips, Mx8000IDT CT scanner in Vienna, Austria, with voxel height and width of 0.47 mm × 0.47 mm, and a slice thickness of 0.75 mm. Modern human CT scans were obtained from various sources and have been described elsewhere^17,49^. Questions regarding the acquisition and possible use of the modern human CT scans can be directed to LNB.

Cranial and maxillary sinuses were digitally segmented and rendered in Amira 5.6^69^ using previously described protocols^17,49,68^. Visual inspection of the fossil scans indicated the presence of foreign materials, including matrix in the nasal cavity, ethmoidal air spaces, and several paranasal sinuses. Processing these scans to remove matrix and segment the maxillary sinuses followed standard semi-automated and manual techniques^70–72^. Additional details regarding the processing of the fossil scans is provided elsewhere^68^. Supplementary Fig. S3 provides the external and internal views of the segmented maxillary sinuses for the Sungir and Mladeč specimens.

**Figure 5:**
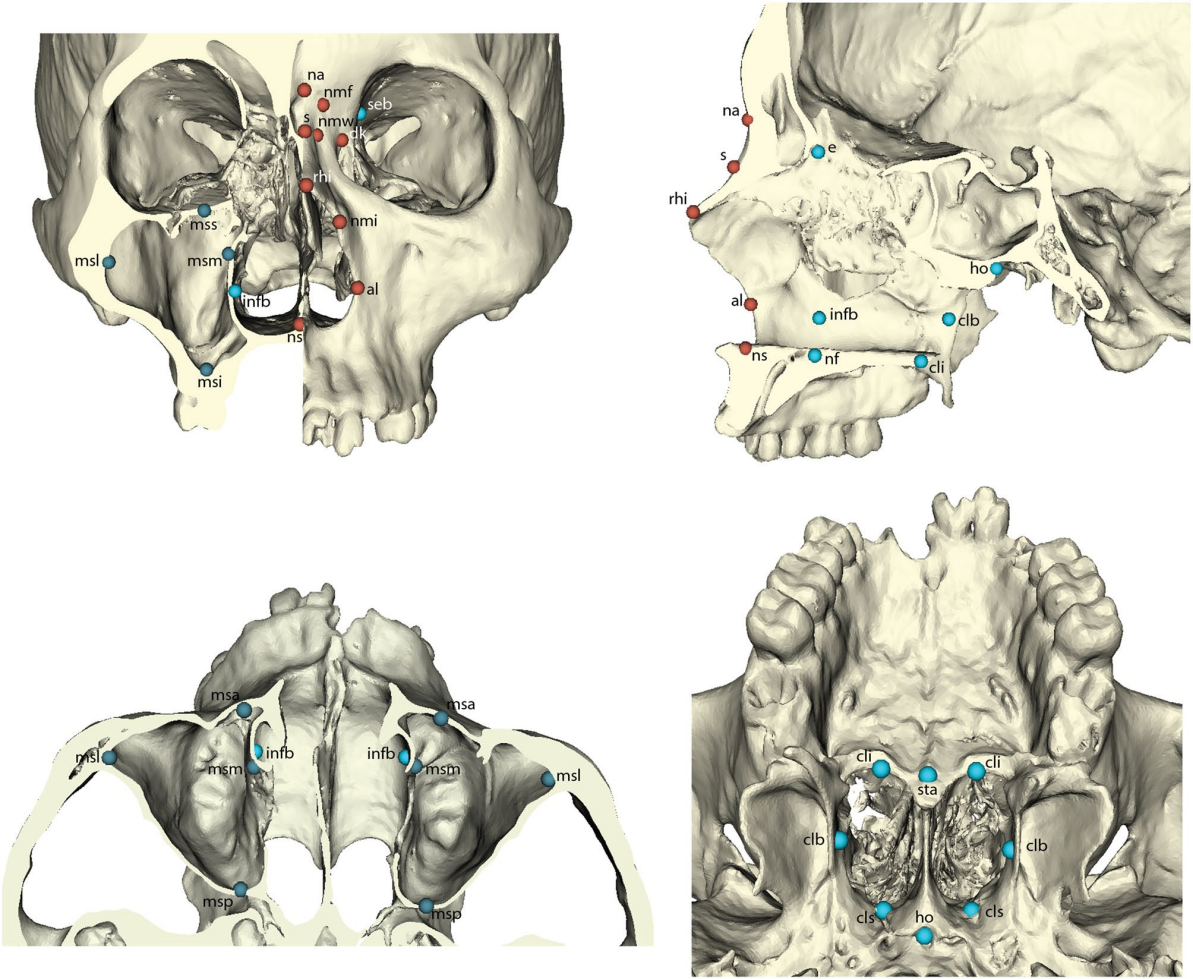
Visualization of landmarks for the external nasal region (colour-coded in red), internal nasal region (light blue), and maxillary sinus region (dark blue). See Table S3 for abbreviations and external facial landmarks not illustrated here.

The rendered cranial and maxillary sinus models were imported into the freeware program 3D-Slicer^73^. A total of 61 landmarks were placed on each digital model, capturing both the external and internal midfacial structures see^17^. These landmarks were further divided into five anatomical regions, which form the basis of the morphometric analyses conducted here: external face, external nose, internal nasal cavity, choanae and maxillary sinuses (see Supplemental Table S3 and Fig. 5 for specific landmarks in each region). To avoid inter-observer error, all landmarks were placed by a single author (LB). Missing landmarks were imputed using thin-plate spline warping^74^; individuals missing more than four landmarks were not included in the study.

Several geographic and climatic variables were collected for each sample. Climatic variables included average minimum temperature for coldest months (TMN), average maximum temperature for warmest months (TMX), average minimum humidity (vapor pressure) for coldest months (HMN), and average maximum humidity (vapor pressure) for warmest months (HMX). Latitude (LAT) and longitude (LON) were determined based on museum records of sample proveniences. Temperature and humidity values were obtained from KNMI Climate Explorer monthly databases (http://climexp.knmi.nl/selectfield_obs2.cgi?id=someone@somewhere). Weather stations were chosen based on the proximity to the samples. Using the monthly field observations database, the climatic values were obtained following the protocols of Maddux et al.^8^. While modern-day climate conditions may not precisely match those of paleoclimates, previous studies indicate that recent climatic data still reflect the environmental pressures that past populations have faced^75,76^. Specific geographic and climatic variables for each modern human sample can be found in Table 1. Geographical distances between groups were obtained following previously published protocols^17^ and accounted for wayward points along the possible migration routes, avoiding migration across large bodies of water and additional environmental barriers^77^.

### Statistical analyses

Data preparation and statistical analyses were carried out in R^78^ using Geomorph^79^, Morpho^80^ and vcvComp^81^ packages. The landmark configurations were registered by Procrustes superimposition^82^ and symmetrised by averaging original and reflected sets of landmarks for each individual. Because of the unbalanced sex composition of the groups, we corrected for sexual dimorphism by subtracting from each individual the sex-specific mean shape and than adding the overall mean^83^. The same treatment was applied to correct for sexual dimorphism in centroid size values. Both fossils were assumed to be male. But we also repeated the analyses without correction, which led to qualitatively similar results.

For each specimen, including the two fossils, we computed the centroid size (CS) of the full landmark configuration along with the centroid sizes of the five anatomical regions (face, external nose, internal nose, choanae and sinuses). Additionally, we calculated relative size values for the five regions by dividing their CS through that of the full configuration of 61 landmarks. Population-specific size distributions were compared in boxplots.

Reduced rank regression (RRR) is a method to decompose the multivariate dependence of one set of variables onto another set of variables into some pairs (or “dimensions”) of linear combinations (latent variables) that best represent this statistical dependence^84–86^. Unlike the more familiar two-block partial least squares analysis (PLS), RRR is not symmetric because the pairs of linear combinations have maximal regression slope (not maximal covariance as in PLS). This difference matters if the independent variables are highly anisotropic (differ in variances and covariances), which can be the case even for variance-standardized variables. For instance, if a combination of independent variables (say, the difference between maximum and minimum humidity) varies considerably less than other combinations (sum of minimum and maximum humidity), it will contribute little to the net covariances and not show up in the first PLS dimension(s). By contrast, RRR maximizes the regression slope, i.e., the average effect of one unit change of the independent variable on the dependent variable, regardless of the variances. E.g., if one unit change in the difference in maximum and minimum humidity had a stronger effect on face shape than one unit change in average humidity, the first dimension of RRR would represent this effect of the differences in max. and min. humidities, even if it varies little across the populations. In other words, we seek the features of face shape (linear combinations of shape variables) that are most responsive to climatic differences, which are not necessarily those features that vary most across populations. As in standard regression models, RRR can be corrected for spatial dependencies by a weighted least squares approach.

More formally, for a sample of size *n*, let **X** be an *n* × *p* matrix of the *p* independent variables (the four climate variables in our application) and **Y** be the *n* × *q* matrix of dependent variables (the 183 shape coordinates). The symmetric *n* × *n* weight matrix **W** represents non-independences among the cases resulting from geographic proximity. Multiple methods have been proposed to estimate or model these spatial autocorrelations (e.g., Dormann et al.^87^). Because the corresponding variogram was basically linear, we simply subtracted the geographic distance between each pair of populations from the maximum of these pairwise distances and divided the result by the same maximum distance. The entries of the resulting weight matrix thus ranged from 0 to 1, with 1 s corresponding to pairs of individuals in the same location. We then computed the *p* × *q* matrix of partial regression coefficients,

$${\mathbf{B}} = \left( {{\mathbf{X}}^{\prime } {\mathbf{W}}^{ - 1} {\mathbf{X}}} \right)^{ - 1} {\mathbf{X}}^{\prime } {\mathbf{W}}^{ - 1} {\mathbf{Y}},$$

and decomposed **B** into its singular vectors and singular values:

$${\mathbf{UDV}}^{\prime } = {\mathbf{B}}.$$

The first column, **u**_1_, of the *p* × *p* matrix **U** is the first loading vector for the climate variables and the first column, **v**_1_, of the *q* × *q* matrix **V** the first loading vector for shape. The second pair of vectors are the loading vectors of the second dimension, and so on. The first singular value, *d*_11_, equals the regression slope of the linear combination **Yv**_1_ on **Xu**_1_, and similarly for the subsequent dimensions.

## Supplementary Information

Supplementary Information
