# Supplementary material for: Respiratory adaptation to climate in modern humans and Upper Palaeolithic individuals from Sungir and Mladeč
Source: Sci Rep. 2021 Apr 12;11:7997. doi: 10.1038/s41598-021-86830-x (PMC8042039; doi:10.1038/s41598-021-86830-x)
Supplement: Supplementary file 1 — Supplementary Information [file 41598_2021_86830_MOESM1_ESM.pdf]

Respiratory adaptation to climate in modern humans and Upper Palaeolithic individuals from Sungir and Mladeč.

Ekaterina Stansfield[1]*, Philipp Mitteroecker[1], Sergey Y. Vasilyev[2], Sergey Vasilyev[3], Lauren N. Butaric[4]

[1] Department of Evolutionary Biology, University of Vienna, Austria.
[2] Moscow State University of Medicine and Dentistry, Russian Federation.
[3] Institute of Anthropology and Ethnography, Russian Federation.
[4] Department of Anatomy, College of Osteopathic Medicine, Des Moines University, United States

Corresponding Author:
Ekaterina Stansfield*
Unit of Theoretical Biology
Department of Evolutionary Biology
University of Vienna, Althanstrasse 14, Vienna 1090, Austria
EMAIL katya.stansfield@gmail.com

Supplementary Information

Reduced Rank Regression of the Shape of the Mid-Face

Table S1. Squared singular values in the analysis of the 61 internal and external mid-facial landmarks.

dimension	Squared singular values	percent of summed squared singular values
1	0.00547	73.19799
2	0.00154	20.58751
3	0.00042	5.670704
4	0.00004	0.543789

CVA of facial landmarks

Figure S1. Canonical variate analysis (CVA) of 61 internal and external mid-facial landmarks. CVA was performed on the first 13 PCs of the shape coordinates (1 less than the smallest group), which described 71% of shape variation. Correct (jack-knifed) classification was obtained in 67.8% of the cases.

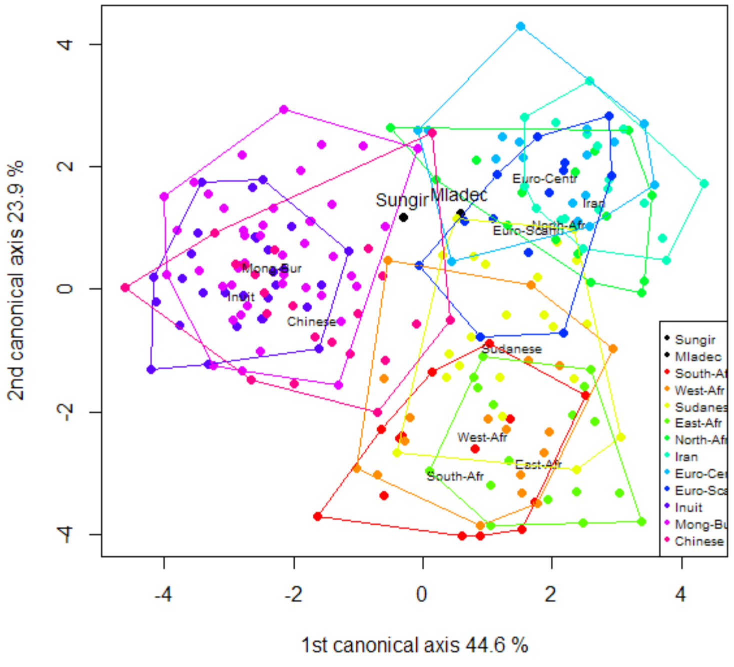

Figure S2. CVA of 61 internal and external mid-facial landmarks: shape deformations associated with the first two canonical axes.

1st canonical axis (dark blue – negative, light blue – positive)	
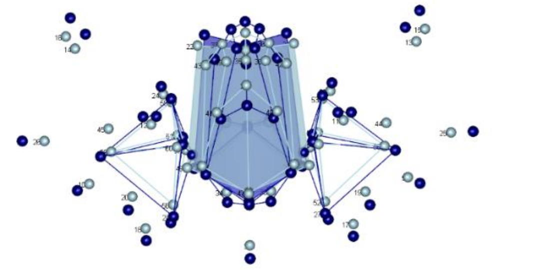 front	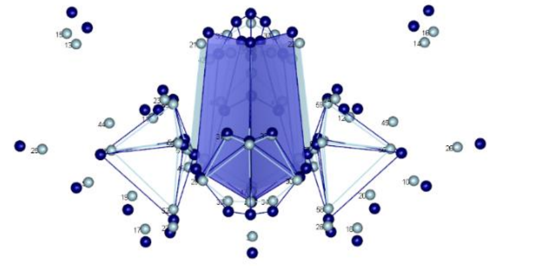 back
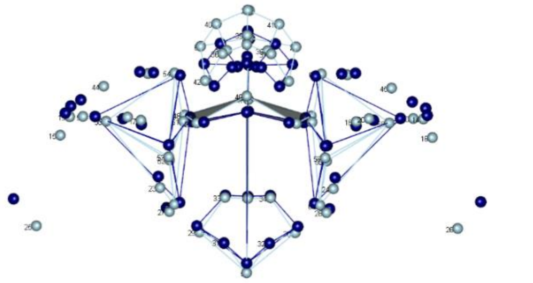 top	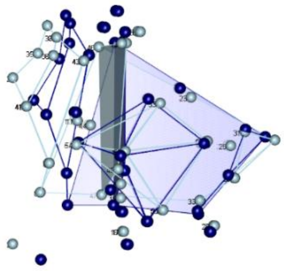 left
2nd canonical axis (dark green – negative, light green – positive)	
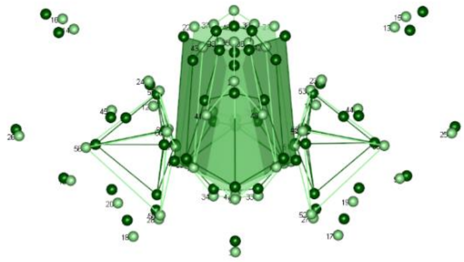 front	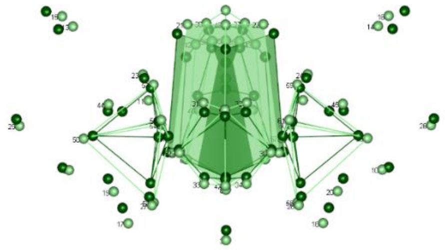 back
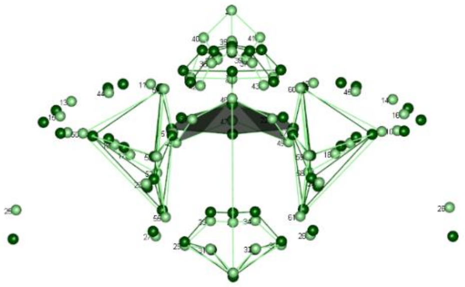 top	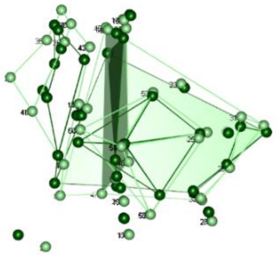 left

Regression of centroid size on climate variables

Table S2. Regression of the centroid sizes on the standardized climate variables.

	Absolute CS						
landmark configuration	intercept	coefficients				R^2	*model p*-value
		TMN	TMX	HMN	HMX		
face	219.85***	-7.644**	-0.0098	4.8687*	1.62219	0.8208	0.01992
external nose	67.465***	-2.9407	-0.0169	0.21861	1.92941	0.586	0.1957
internal nose	82.608***	-2.5008	0.9582	-0.1601	1.2465	0.419	0.4427
choanae	39.874***	-1.7761	0.3278	-0.5941	*1.5989*	0.5769	0.2068
sinuses	101.17***	-1.9282	0.8812	-1.4272	2.0448	0.2926	0.6647
	Relative CS						
landmark configuration	intercept	coefficients				R^2	*model p-*value
		TMN	TMX	HMN	HMX		
face	n/a	n/a	n/a	n/a	n/a	n/a	n/a
external nose	0.3072***	0.0004*	-0.0001	-0.0121	0.00842	0.5237	0.2778
internal nose	0.37551***	-0.002*	0.00509	-0.0063	0.00136	0.5538	0.2364
choanae	0.18058***	-0.002*	0.00161	-0.0068	0.0054	0.394	0.4856
sinuses	0.45989***	0.0013*	0.0083	-0.0132	0.00558	0.6564	0.1205

*** significance of individual predictor values at 0.001 level

** significance of individual predictor values at 0.05 level

* significance of individual predictor values at 0.1 level

Material and Methods: Supplementary Information

Figure S3. Internal Views of Sungir (top) and Mladeč (bottom). The left maxillary sinus is segmented and highlighted in grey in each skull.

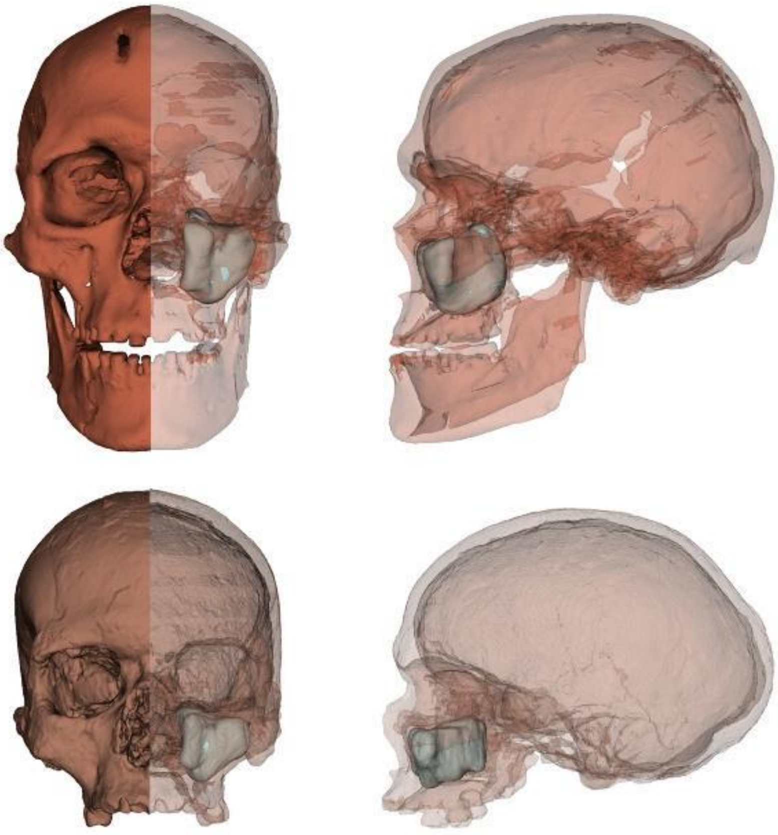

Table S3. Landmarks utilized in the current study, along with the cranial region they are assigned to: face, external nose, internal nose, or choanal region. Abbreviations correspond to Figure 5.

Landmark Name (abr.)	Region	Description
Prosthion (pr)	Face	Midline, most anterior point on maxillary alveolar process
Zygomaxillare anterior (zma)	Face	Paired points on inferior/anterior zygomaticomaxillary suture
Zygoorbitale (zmo)	Face	Paired points on superior zygomaticomaxillary suture at inferior orbital rim
Frontomalare orbitale (fmo)	Face	Paired points on the frontomalar suture along medial surface of lateral orbital rim

Frontomalare temporale (fmt)	Face	Paired points on the frontomalar suture along lateral surface of lateral orbital rim
Alveolare (alv)	Face	Paired points on most lateral external alveolar process
Maximum maxillary curvature (mmc)	Face	Paired points on the maximum curvature between upper alveolar process and zygomaxillary suture (Lahr, 1992)
Maxillary tuberosity superior (mts)	Face	Paired points at most superior aspect of posterior maxillary body, typically at/near constriction of the inferior orbital fissure
Zygotemporal inferior (zti)	Face	Paired points at the inferior aspect of zygotemporal suture
Maxillary tuberosity inferior (mti)	Face	Paired points at the most posterior aspects of the maxillary tuberosity, at the alveolar process
Dacryon (dk)	Face	Paired points at the apex of the lacrimal fossa, adapted from
Orbitale (or)	Face	Paired points at the most inferior point of each inferior orbital rim
Nasion (na)	Ext Nose	Midline point on fronto-nasal suture
Rhinion (rhi)	Ext Nose	Midline point at inferior end of nasals
Alare (al)	Ext Nose	Paired points on the most lateral aspects of the anterior nasal piriform aperture
Nasospinale (ns)*	Ext & Int Nose	Midline point on floor of the piriform nasal aperture (adapted from Martin, 1928)
Superior Ethmoid (seb)*	Ext & Int Nose	Paired points along the frontal-ethmoidal suture on the medial orbital wall, as when taking superior ethmoidal breadth (Following Franciscus, 1995)
Sellion (s)	Ext Nose	Midline point of deepest curvature between nasion and rhinion; also known as subnasion
Nasomaxillary frontale (nmf)	Ext Nose	Paired points at junction of the frontonasal and nasomaxillary sutures
Nasomaxillary minimum (nmw)	Ext Nose	Paired points on nasomaxillary suture, as when taking minimum (simotic) nasal breadth
Nasomaxillary inferior (nmi)	Ext Nose	Paired points at the most inferior aspect of the nasomaxillary suture, where it meets the piriform aperture

Ethmoidale (e)	Int Nose	Midline point at junction of ethmoid and frontal bones on nasal roof, as when taking maximum internal nasal height with the *nasal floor (nf)* (Butaric, 2015)
Nasal floor (nf)	Int Nose	Midline point on nasal floor directly below ethmoidale, as when taking maximum internal nasal height with *ethmoidale (e)* (Butaric, 2015)
Inferior nasal meatus (inf)	Int Nose	Paired points at the lateral aspects of the inferior nasal meatus, as when taking maximum internal nasal breadth (Butaric, 2015)
Staphylion (sta)*	Int Nose & Choanae	Midline point on hard palate, crossed by tangent line across curves of posterior palatal margin
Hormion (ho)*	Int Nose & Choanae	Midline point where vomer intersects the sphenoid
Choanal/posterior alare (clb)	Choanae	Paired points on most lateral aspects of choanal aperture, on medial surface of medial pterygoids; as when taking maximum choanal breadth
Choanal superior (cls)	Choanae	Paired points on superior aspect of sphenoid, as when taking choanal height; also known as choanal apex or roof
Choanal inferior (cli)	Choanae	Paired points on the posterior edge of palate, as when taking choanal height; also known as choanal base or floor
Lateral maxillary sinus (msl)	Sinus	Paired points on most lateral aspects (apex) of each maxillary sinus
Medial maxillary sinus (msm)	Sinus	Paired points on each medial maxillary sinus wall; as when taking sinus breadth from the apex, parallel to Frankfort plane
Superior maxillary sinus (mss)	Sinus	Paired points on each maxillary sinus roof; as when taking maximum sinus height perpendicular to Frankfort plane
Inferior maxillary sinus (msi)	Sinus	Paired points on each maxillary sinus floor; as when taking maximum sinus height perpendicular to Frankfort plane
Anterior maxillary sinus (msa)	Sinus	Paired points on each maxillary sinus anterior wall; as when taking maximum sinus length/depth parallel to Frankfort plane
Posterior maxillary sinus (msp)	Sinus	Paired points on each maxillary sinus posterior wall; as when taking maximum sinus length/depth parallel to Frankfort plane

* indicates a landmark that was used in more than one morphological region.
